# Rare allele of *HvLox*-*1* associated with lipoxygenase activity in barley (*Hordeum vulgare* L.)

**DOI:** 10.1007/s00122-014-2362-3

**Published:** 2014-09-12

**Authors:** Ganggang Guo, Dawa Dondup, Xingmiao Yuan, Fanghong Gu, Deliang Wang, Fengchao Jia, Zhiping Lin, Michael Baum, Jing Zhang

**Affiliations:** 1 Key Laboratory of Crop Germplasm Resources and Utilization (Ministry of Agriculture), The National Key Facility for Crop Gene Resources and Genetic Improvement, Institute of Crop Science, Chinese Academy of Agricultural Sciences, Beijing, 100081 China; 2 Tibet Academy of Agricultural and Animal Husbandry Sciences, Lhasa, 850032 China; 3 China National Research Institute of Food and Fermentation Industries, Beijing, 100027 China; 4 Technical Research Center of Beijing Yanjing Brewery Group Co. Ltd, Beijing, 101300 China; 5 International Center for Agricultural Research in the Dry Areas, Amman, 11195 Jordan

## Abstract

*****Key message***:**

**Identification and allele-specific marker development of a functional SNP of** ***HvLox*** **-** ***1*** **which associated with barley lipoxygenase activity.**

**Abstract:**

Improving the stability of the flavor of beer is one of the main objectives in breeding barley for malting, and lipoxygenase-1 (LOX-1) is a key enzyme controlling this trait. In this study, a modified LOX activity assay was used for null LOX-1 mutant screening. Four barley landraces with no detected level of LOX-1 activity were screened from 1,083 barley germplasm accessions from China. The genomic sequence diversity of the *HvLox*-*1* gene of the four null LOX-1 Chinese landraces was compared with that of a further 76 accessions. A total of 104 nucleotide polymorphisms were found, which contained 83 single-nucleotide polymorphisms (SNPs), 7 multiple-nucleotide polymorphisms, and 14 insertions and deletions. Most notably, we found a rare C/G mutation (SNP-61) in the second intron which led to null LOX-1 activity through an altered splicing acceptor site. In addition, an allele-specific polymerase chain reaction marker was developed for the genotyping of SNP-61, which could be used in breeding programs for barley to be used for malting. The objective was to improve beer quality.

**Electronic supplementary material:**

The online version of this article (doi:10.1007/s00122-014-2362-3) contains supplementary material, which is available to authorized users.

## Introduction

The metabolism products of lipid degradation seriously affect the quality of cereals and their processed products during storage (Garbus et al. 2009; Wang et al. 2008; Zhang et al. 2007). In the lipid degradation pathway, the polyunsaturated fatty acid substrate is converted to 9-hydroperoxide (9-HPOD) and 13-hydroperoxide (13-HPOD) by the dioxygenase effect of lipoxygenase (Kuroda et al. 2003; Kuroda et al. 2005). Subsequently, 9-HPOD is converted to trans-2-nonenal (T2N) and trihydroxy octadecenoic acids (THODs) by HvHPL2 and its isoenzymes. At the same time, 13-HPOD is metabolized to hexanal (Feussner and Wasternack 2002). T2N was identified as being responsible for the cardboard-like flavor in beer (Hambraeus and Nyberg 2005), and it is generated and accumulated during beer storage (Kuroda et al. 2005; Vanderhaegen et al. 2006). In addition, THOD also has an adverse effect on foam stability and the flavor of beer (Kaneda et al. 2001; Kobayashi et al. 2002). Therefore, there has been extensive screening of artificial or natural variations for null- or reduced-LOX barley mutants (Hirota et al. 2005; Oozeki et al. 2007; Patent US7420105 B2).

As a key enzyme in the conversion of linoleic acid to THODs in the lipid degradation pathway, LOX is encoded by a gene family in plants. For example, three Lox genes were identified in barley—*Lox*-*1* (*LoxA*), *Lox*-*2* (*LoxC*), and *Lox*-*3* (*LoxB*) (Rouster et al. 1997, 1998; van Mechelen et al. 1999; van Mechelen et al. 1995). In beer production, LOX-1 catalyzes the formation of 9-HPOD while LOX-2 catalyzes the formation of 13-HPOD. LOX-3 is considered irrelevant to beer quality given its low expression level in barley kernels and unclear product specificity (van Mechelen et al. 1999). Collectively, LOX-1 is considered the main contributor to malt LOX activity (Doderer et al. 1992; Kuroda et al. 2003; Yang and Schwarz 1995). To date, several artificial and natural mutants of null LOX-1 activity have been screened out and used for the breeding of flavor-stable malting barley. Forced aging tests on the null LOX beer produced also demonstrated its improved flavor stability through reduced T2N content (Hirota et al. 2005, 2006).

Genetic improvement, extending the stability of beer flavor and its shelf life has been one of the main targets in breeding barley for malting. We sought to identify the related gene and develop the gene’s molecular markers for the quality characteristics useful to barley breeders. Thus, in the present study, 1,083 barley landrace accessions were screened for null LOX-1 activity mutants. Additionally, 77 barley landraces, including undetectable LOX activity mutants and randomly selected genotypes, were sequenced and analyzed for the diverse alleles/haplotypes and key single-nucleotide polymorphisms (SNPs) affecting LOX-1 activity. The mechanism of LOX-1 activity loss in the mutant is also discussed.

## Materials and methods

### Plant materials

In the process of screening for null LOX-1 activity barley, 1,083 barley landraces from the National Genebank of China were purified by a single plant harvest and individually milled with a Perten™ laboratory mill (PLM3100/C, 0.8 mm standard sieve). Crude homogenates were prepared by mixing and vigorously vortexing 0.1 g of barley powder in 1 mL of ice cold water in a 1.5 mL Eppendorf tube, and then centrifuging at 3,000 rpm for 2 min at 4 °C. Then, 50 μL of supernatant was aspirated for the lipoxygenase enzyme activity assay.

### Lipoxygenase assay

Chemical preparation and the activity analysis of the linoleic hydroperoxide reaction of LOX were performed using a modified DMAB–MBTH assay as described in Anthon and Barrett (2001). For the modified rapid assay, a fresh working solution A was prepared by mixing 10 mL of DMAB solution (20 mM DMAB, 100 mM phosphate buffer, and pH 6.0), 0.4 mL of linoleic acid substrate (25 mM linoleic acid stock), and 9.6 mL of water. Solution B was also freshly prepared by mixing 0.4 mL of MBTH (10 mM), 0.4 mL of hemoglobin (5 mg/mL), and 19.2 mL of water. All assays of linoleic hydroperoxide were carried out at room temperature (20–25 °C). The sample was incubated initially with 0.5 mL of solution A for 20 min and 0.5 mL of solution B was added subsequently. After an additional 10 min, the reaction was terminated by adding 0.5 mL of 1 % (w/v) sodium lauryl sulfate (SLS). Blank tests were performed by adding SLS before the reaction. For samples with normal LOX-1 activity, stable dark blue products were quickly produced. The presence or absence of dark blue products was used as a rapid visual determination of LOX-1 activity.

### Nucleotide extraction

Barley plants were grown in an illuminated incubator under the following conditions: 22 °C for 16 h of light and 18 °C for 8 h of darkness. Two-week-old seedlings were harvested individually and frozen in liquid nitrogen and then stored at −80 °C for DNA and RNA extraction. DNA and RNA were isolated and purified with the DNeasy™ and RNeasy™ plant mini kit (Qiagen) according to the manufacturer’s instructions.

### Allele mining and cDNA cloning

*HvLox*-*1* containing contigs of cvs Morex, Bowman, and Barke were obtained from the assembled whole-genome shotgun barley genomic sequence database in the Leibniz Institute of Plant Genetics and Crop Plant Research (IPK) (http://webblast.ipk-gatersleben.de/barley/) using a previously reported barley *Lox*-*1* sequence (GenBank accession: U83904.1) as a query (International Barley Genome Sequencing Consortium 2012). Primers were generated with Primer3 online software (Rozen and Skaletsky 2000). Five pairs of overlapping primers (Lox1.1–Lox1.5) were designed to cover the 5,884-bp genomic region of *HvLox*-*1* (including 1,500 bp upstream and 204 bp of 3′ UTR), and two pairs of primers (Lox1.FL.1 and Lox1.FL.2) were designed for cDNA cloning (Table S1).

A total of 77 barley germplasms were sequenced for allele mining and haplotype analysis. These included 73 LOX-1 enzyme activity positive lines from the major barley-growing regions of China and four null LOX-1 landraces (ZDM00279, ZDM00626, ZDM00677, and ZDM05300). Moreover, all null LOX-1 lines were confirmed with a second sequencing. Details of accession names, their haplotypes, row type, and origin are listed in Table S2.

Two micrograms of total RNA were reverse transcribed to cDNA in 20 μL reactions containing 50 mM Tris–HCl (pH 8.3), 75 mM KCl, 3 mM MgCl_2_, 10 mM DTT, 50 μM dNTPs, 200 U M-MLV reverse transcriptase (Promega), and 1 μg of oligo-dT primer. Reverse transcription was performed for 60 min at 37 °C with a final denaturation step at 95 °C for 5 min.

The amplification of the *HvLox*-*1* gene fragment was performed using Pfu-DNA polymerase (TIANGEN, China). The PCR conditions were 35 cycles at 95 °C for 30 s, 58 °C for 30 s, and 72 °C for 2 min, followed by a 7-min extension at 72 °C. The PCR product was purified and sequenced directly using an ABI3730 sequencer. The haplotypes corresponding to full-length cDNA of *HvLox*-*1* from eight lines (ZDM00470, ZDM00797, ZDM03953, ZDM03595, ZDM00279, ZDM00626, ZDM00677, and ZDM05300) were amplified and sequenced. Nucleotide sequences were assembled with PHRAP software (Bastide and McCombie 2007) and manually edited using BioEdit 7.0 (Hall 1999). Multiple-sequence alignments were carried out with the ClustalW v2.1 program (Larkin et al. 2007). The allelic haplotypes and sliding-window plot (window length 100 bp, step size 25 bp) of nucleotide diversity (*π*) of the *HvLox*-*1* gene were defined and generated by DnaSP 5.10 (Librado and Rozas 2009). A neighbor-joining (NJ) tree of seven haplotypes of *HvLox*-*1* was constructed using MEGA 6.0 with a 1,000 times bootstrap (Tamura et al. 2013). All nucleotide variations, amino acids, and predicted regulatory motif changes are shown in Table S3.

### Allele-specific PCR for functional SNP genotyping

An allele-specific PCR fragment length polymorphism analysis (AS-PCR) assay was used for SNP validation. Two pairs of primers were developed for the identification and genotyping of SNP-61 as described in Wangkumhang et al. (2007). A pair of primers, RNF/Lox1.FL.1R, was designed for ‘C’ allele genotyping with a 320 bp specific PCR amplicon. The primer pairs Lox1.FL.2F and RNR were able to discriminate the ‘G’ allele from null LOX-1 barley lines for which a 720-bp specific PCR product was generated. A 10 bp (CACTAGTGAT), randomly selected flanking sequence from a common cloning vector was added to the 5′-end of the RNR primer to improve PCR stability. All these primer pairs were multiplexed in a single-tube PCR assay to assess the allelic status at SNP-61 (Table S1; Fig. 4a). The amplification of the allele-specific gene fragment was performed under PCR conditions of 35 cycles at 95 °C for 30 s, 65 °C for 30 s, and 72 °C for 30 s; followed by a 5-min extension at 72 °C. The PCR products were separated using a 1 % agarose gel.

## Results

### Characterization of LOX-1 activity in Chinese barley landraces

**Fig. 1:**
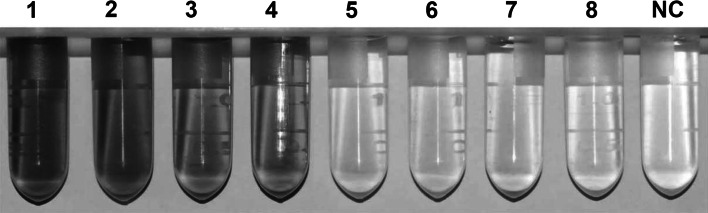
Identification of null LOX-1 activity landrace lines. LOX-1 activity positive lines: *1* (H2): ZDM00470; *2* (H3): ZDM00797; *3* (H4): ZDM03595; *4* (H5): ZDM03953. Identified null LOX-1 activity barley landrace lines: *5* (H6): ZDM00279; *6*–*8* (H7): ZDM00626, ZDM00677, and ZDM05300; *NC* negative control

A total of 1,083 Chinese barley (*Hordeum vulgare* L.) landraces were analyzed using a rapid visual determination of LOX-1 activity assay. Four barley landraces (ZDM00279, ZDM00626, ZDM00677, and ZDM05300) showed no significant LOX-1 activity in silenced seeds (Fig. 1), which was further confirmed by a malted barley analysis (data not shown). The four barley landraces without LOX-1 activity originated from Shan-Dong and He-Nan Provinces of China (Table S2).

### Allelic variation and sequence diversity of *HvLox*-*1*

**Fig. 2:**
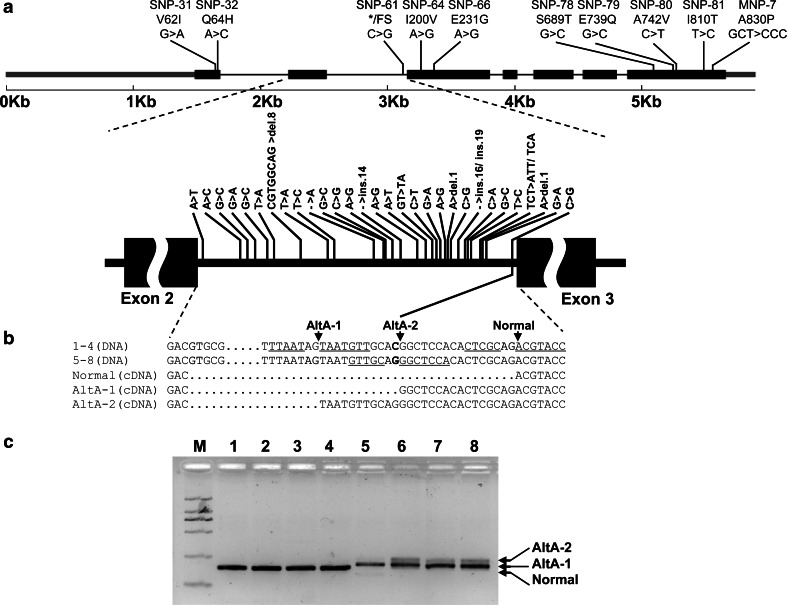
Allelic diversity of *HvLox*-*1.* **a** Schematic of the exon–intron structure of the *HvLox*-*1* gene. The positions of nine non-synonymous amino acid changes are shown above the gene structure and the SNP-61 that induced a premature stop codon and/or frame-shift of HvLOX-1 is shown in *red* (corresponding to Table 1). The nucleotide variations of intron 2 are shown below (corresponding to Table 2). **b** SNP-61 in the second intron causing alternative splicing of *HvLox*-*1*. SNP-61 affecting an intron–exon splice junction and resulting in a premature termination (*5*–*8*) and frame-shift (*6*–*8*) in the coding sequence. The positions of the SNP-61 natural mutant alleles in the genomic DNA and processed cDNA sequence alignment and two types of alternative splicing events are shown below the gene structure. *1*–*8*: as for Fig. 1. **c** Alternative splicing induced cDNA length polymorphism of *HvLox*-*1*. *1*–*8*: as for Fig. 1. In contrast to the normal splicing form of intron 2 in *1*–*4* (H2–H5), *5* (H6) is the only AltA-1 event shown, and *6*–*8* (H7) are shown as the two types of alternative splicing events, AltA-1 and AltA-2 (color figure online)

**Fig. 3:**
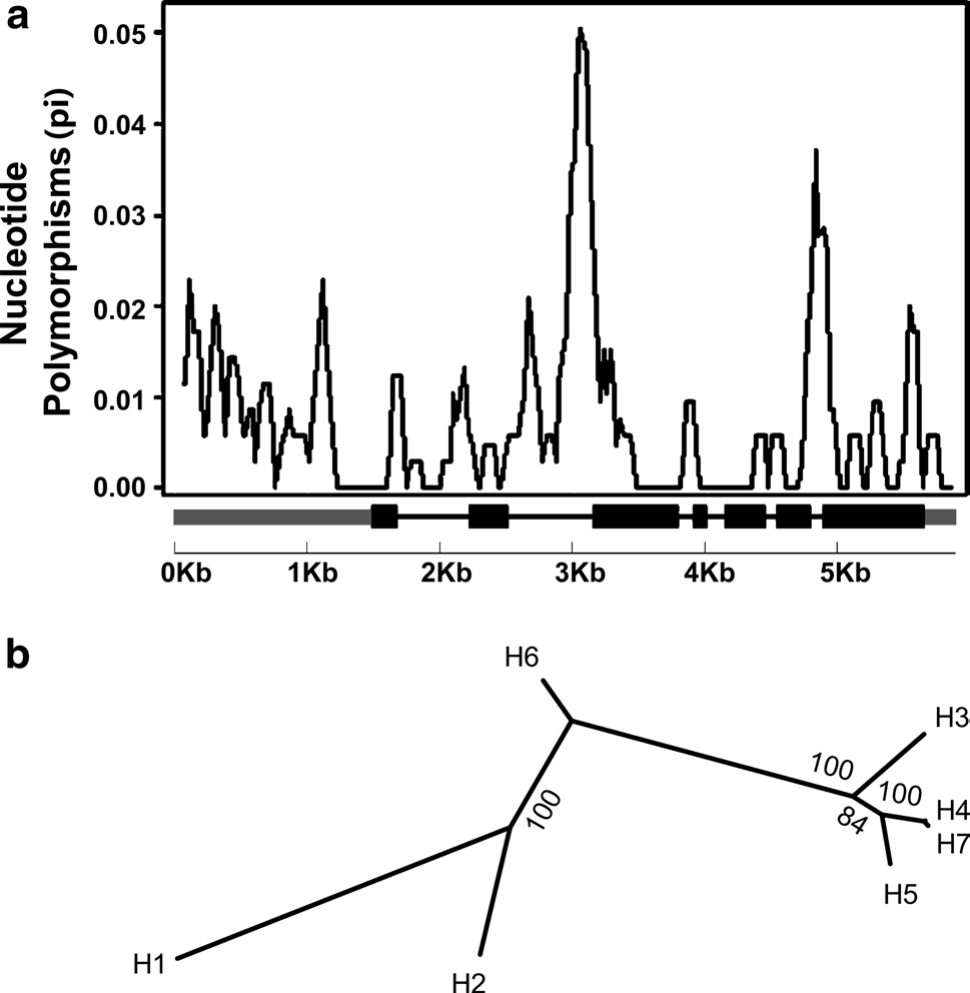
Sliding-window analysis and neighbor-joining tree of the seven haplotypes of *HvLox*-*1.* **a** Sliding-window representation of the *HvLox*-*1* gene polymorphism sites, which correspond to the exon–intron structure of the seven haplotypes, was executed using DnaSP 5.10, the window length was 100 bp, and the step size was 25 bp. **b** A neighbor-joining (NJ) tree of seven haplotypes of the *HvLox*-*1*. The NJ tree of the seven haplotypes was developed using the neighbor-joining algorithm of MEGA 6.0

The nucleotide variation analysis of *HvLox*-*1* was carried out using the contig sequences of three non-Chinese barley cultivars (cvs Morex, Bowman, and Barke) and 77 sequenced Chinese barley landraces (including four null LOX-1 activity lines). A total of 104 varied DNA polymorphisms—including 83 SNPs, 7 multiple-nucleotide polymorphisms (MNPs) and 14 insertions and deletions (InDels)—were observed in the 5,884-bp genomic region of *HvLox*-*1*, with an average frequency of ~17 DNA polymorphisms for every kilo base-pair (*π* = 0.01209). The nucleotide diversity in the non-coding region was much higher than in the exons. There were 23 nucleotide polymorphisms in the exons (2,589 bp) and 43 in the introns (1,464 bp) of *HvLox*-*1* (Table S3). Six out of seven multinucleotide polymorphisms (MNPs) and all of the 14 InDels were located in the non-coding region. Also, nine non-synonymous SNPs were located in three exons (exons 1, 3, and 7) (Fig. 2a; Table 1). In addition, the second intron of the gene contained 30 nucleotide variations in the 634 bp region, with about half of these being concentrated in a 100 bp region (1,500–1,600 bp) (Fig. 2a; Table 2). Sliding-window analysis of the polymorphism pattern across the *HvLox*-*1* gene also revealed that the nucleotide diversity in the coding region was far less than in the exons and promoter region. Notably, the peak of nucleotide polymorphism (pi) was located at the second intron of the *HvLox*-*1* gene, which demonstrated that intron 2 is the high-density area of DNA polymorphisms (Fig. 3a).

**Table 1:** Protein diversity of HvLOX-1

Genomic region	Exon 1			Exon 2		Intron 2	Exon 3					Exon5	Exon 6					Exon 7								Haplotype	Number of accessions
Protein type	SNP-30	SNP-31	SNP-32	SNP-38	SNP-39	*SNP-61*	SNP-62	SNP-63	SNP-64	SNP-65	SNP-66	SNP-69	SNP-71	SNP-72	SNP-73	SNP-74	SNP-75	SNP-76	SNP-77	SNP-78	SNP-79	SNP-80	SNP-81	MNP-7	SNP-82		
HvLOX1-1	C	G	A	G	A	C	G	C	A	C	A	C	G	C	T	C	G	T	G	G	G	C	T	GCT	T	H1	3
HvLOX1-2	C	A	A	G	C	C	A	C	A	T	G	C	A	C	T	C	G	T	G	G	C	T	T	CCC	C	H2	51
HvLOX1-3	A	G	C	C	C	C	A	C	G	C	G	C	G	T	C	T	C	C	G	C	G	C	T	GCT	T	H3, H4	5, 8
HvLOX1-4	A	G	C	C	C	C	G	G	G	C	G	T	G	T	C	T	C	C	A	G	G	C	C	CCC	C	H5	9
HvLOX1-5	A	G	C	C	C	*G*	G	G	G	C	G	T	G	T	C	T	G	T	G	G	C	T	T	CCC	C	H6	1
HvLOX1-6	A	G	C	C	C	*G*	G	G	G	C	G	T	G	T	C	T	C	C	G	C	G	C	T	GCT	T	H7	3
aa position	53	62	64	107	154	*164*	176	178	200	226	231	484	572	580	582	589	600	621	630	689	739	742	810	830	832		
aa change	No (T)	**V/I**	**Q/H**	No (G)	No (R)	*/FS	No (P)	No (R)	**I/V**	No (R)	**E/G**	No (Y)	No (E)	No (F)	No (L)	No (Y)	No (P)	No (L)	No (A)	**S/T**	**E/Q**	**A/V**	**I/T**	**A/P**	No (F)		

The first two rows indicate the position of the SNPs; the last row reveals the amino acid changes. Bold indicates non-synonymous SNPs. Six types of HvLOX1 protein are identified. SNP-61 (in italic) indicates that HvLOX1–5 and HvLOX1–6 were permutation and frame-shift types. The numbers in the right hand column are the numbers of cultivars represented in every protein type

**Table 2:** Natural allelic variation in the intron 2 of *HvLox*-*1*

Haplotype	SNP-40	SNP-41	SNP-42	SNP-43	SNP-44	SNP-45	InDel-4	SNP-46	SNP-47	InDel-5	SNP-48	SNP-49	SNP-50	InDel-6	SNP-51	SNP-52	MNP-5	SNP-53	SNP-54	SNP-55	InDel-7	SNP-56	InDel-8	SNP-57	SNP-58	SNP-59	MNP-6	InDel-9	SNP-60	SNP-61
H1	A	A	G	G	G	T	CGTGGCAG	T	T	.	G	C	A		A	A	GT	C	G	A	A	C		C	G	T	TCT	A	G	C
H2	T	C	C	A	C	T		A	C	.	G	C	G		G	A	GT	C	G	G	A	C		C	G	T	ATT	A	A	C
H3	T	C	C	G	C	T	CGTGGCAG	T	T	.	G	C	A		G	A	GT	T	G	G	A	G	ins.16	A	G	C	TCT	A	A	C
H4	T	C	G	G	G	A		T	T	A	C	G	A	ins.14	G	T	TA	T	A	G		G	ins.19	A	C	C	TCA		A	C
H5	T	C	G	G	G	A		T	T	A	C	G	A	ins.14	G	T	TA	T	A	G		G	ins.19	A	C	C	TCA		A	C
H6	T	C	G	G	G	A		T	T	A	C	G	A	ins.14	G	T	TA	T	A	G		G	ins.19	A	C	C	TCA		A	**G**
H7	T	C	G	G	G	A		T	T	A	C	G	A	ins.14	G	T	TA	T	A	G		G	ins.19	A	C	C	TCA		A	**G**

Bold indicates the SNP shared by null-LOX1 barley haplotypes H6 and H7

**Fig. 4:**
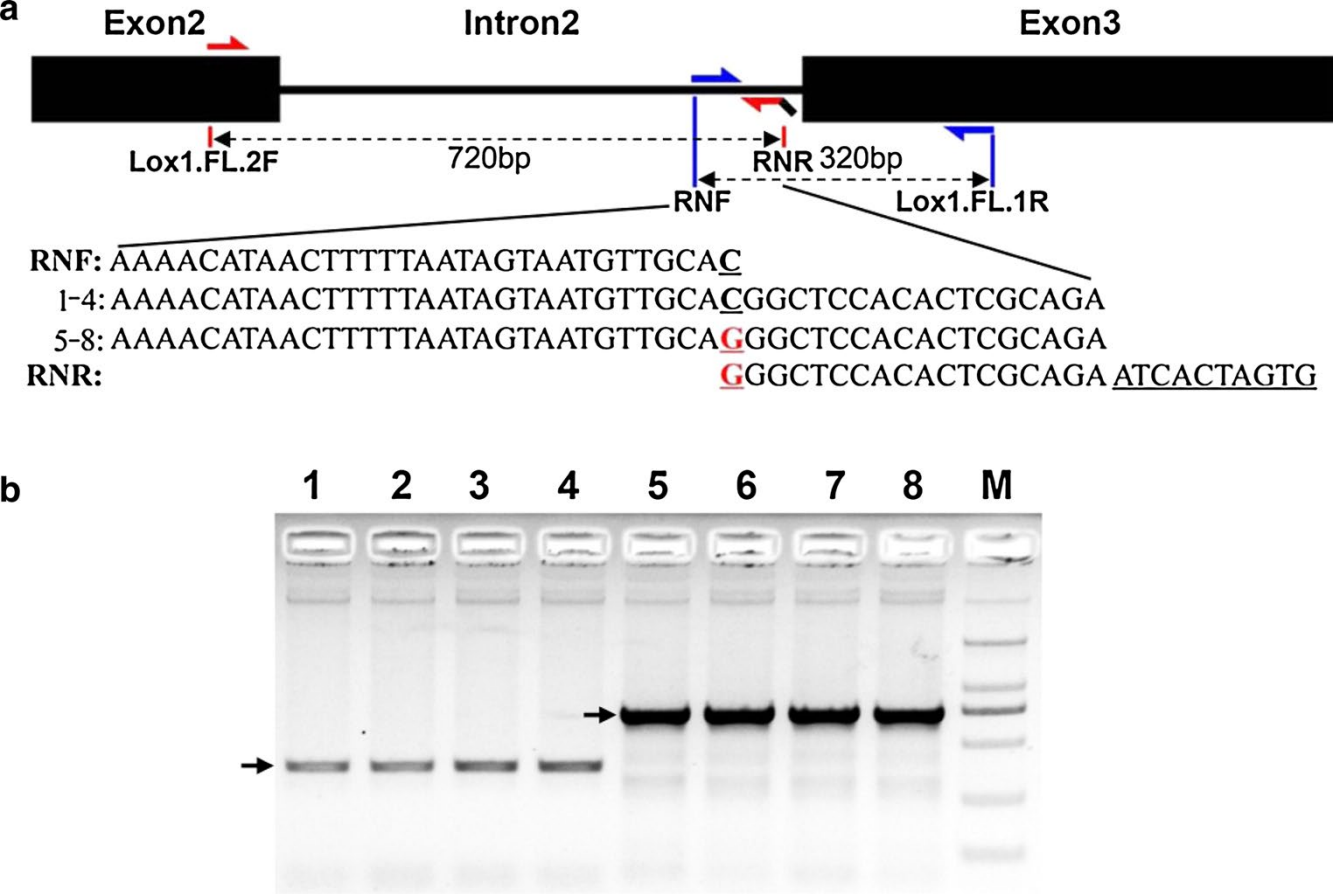
Allele-specific PCR marker developing and genotyping. **a** Schematic illustration of the development of allele-specific PCR primers for SNP-61. A pair of primers (RNF/Lox1.FL.1R) was designed for ‘C’ allele genotyping with a 320-bp specific PCR amplicon. The primer pairs Lox1.FL.2F and RNR were able to discriminate the ‘G’ allele from the null-LOX1 barley lines for which the primer pairs were generated as a 720-bp specific PCR product. All these primer pairs were multiplexed in a single-tube PCR assay to assess the allelic status at SNP-61. **b** Gel analysis can clearly distinguish the allelic status of SNP-61. *Lanes* *1*–*8* are the same as Fig. 1. *Two* *arrows* indicate the 720-bp (G allele) and 320-bp (C allele) specific PCR products separately; *M* DL2000 ladder

A total of 34 nucleotide polymorphisms, containing 29 SNPs, three MNPs and two InDels, were found in the 1.5 kb promoter region of *HvLox*-*1*. The *HvLox*-*1* promoters in Chinese barley landraces were distinctly different from those in the American and European barley cultivars. Based on the SNP changes in the promoter of each haplotype, there was a clear division into two types (H1 and H2–H7) (Table S3).

### Haplotype analysis and identification of critical variation associated with null LOX-1 activity

The 80 barley accessions analyzed contained seven haplotypes according to the detected nucleotide polymorphisms. The American and European cvs Morex, Bowman, and Barke were defined as haplotype H1. Six haplotypes (H2–H7) were constructed from the remaining 77 Chinese barley landrace lines. A total of 51 landraces were identified as haplotype H2; these landraces were widely distributed in the major barley-growing regions of China. Five landraces were assigned to haplotype H3, eight to H4, and nine to H5. Of the four barley accessions with null LOX-1 activity, ZDM00279 was assigned to H6 and ZDM00626, ZDM00677, and ZDM05300 to H7. These latter shared the same SNP (SNP-61, C/G) and originated from He-Nan and Shan-Dong Provinces (Table S2). Haplotypes H2, H3, and H6 partly contained H1 SNP alleles and were quite distinct from haplotypes H4, H5, and H7, which showed completely different SNP alleles to H1 (Table S3) and were grouped into one phylogenetic sub-tree (Fig. 3b). Thus, SNP-61 was considered as the candidate variation that may affect the function of HvLOX-1 protein. Two clades were detected through a phylogenetic analysis of *HvLox*-*1* (Fig. 3b), which corresponded with the promoter analysis result and with the polyphyletic domestication division of cultivated barley (Dai et al. 2012).

To further investigate the relationship of SNP-61 to null LOX-1 activity, the *HvLox*-*1* cDNAs from all haplotypes were sequenced and analyzed. A total of 25 SNPs were detected in the coding region, including nine non-synonymous SNPs, 15 synonymous SNPs, and one SNP (SNP-61, C/G). These SNPs were divided into two types of altered splicing acceptor sites (AltA)—AltA-1 and AltA-2 (Table 1; Fig. 2b, c)—corresponding to six protein types. The cvs Morex, Bowman, and Barke shared the same protein type HvLOX1-1, while H2 (ZDM00470, ZDM03692, ZDM03956, and ZDM08055) encoded the protein type HvLOX1-2. Haplotypes H3 (ZDM00797) and H4 (ZDM03595) shared the same protein type HvLOX1-3. H5 (ZDM00586, ZDM00691, ZDM00028, and ZDM03953) encoded the HvLOX1-4 protein type. Haplotype H6 (ZDM00279) encoded the protein type HvLOX1-5, which corresponded to the AltA form of AltA-1. Haplotype H7 (ZDM00626, ZDM00677, and ZDM05300) generated two protein types, HvLOX1-5 and HvLOX1-6, corresponding to the two AltA types, AltA-1 and AltA-2. Among the six protein types of HvLOX1 found, the four types of HvLOX1-1, HvLOX1-2, HvLOX1-3, and HvLOX1-4 encoded a complete 862-aa protein (Table 1). The other two types, HvLOX1-5 and HvLOX1-6, were truncated to 309 and 163 aa, respectively, since the alternative splicing induced frame-shift and premature termination in *HvLox*-*1* (Fig. 2b, c; Table 1). Compared with the other four HvLOX1 types, the HvLOX1-5 and HvLOX1-6 barley landraces showed null LOX-1 activity. The result revealed that the protein diversity of HvLOX-1 induced by AltA was critical to the variation of enzyme activity. SNP-61 was identified as the critical variant for altering the barley LOX-1 activity.

## Discussion

Barley HvLOX-1 involvement in lipid degradation and the formation of beer-deteriorating substances have been studied previously (Kuroda et al. 2003). Furthermore, null LOX beer has become a highly desirable product, since it has significant stability of both flavor and foam, which allows the beer to remain fresh for longer. So far, several natural and artificial null LOX-1 barley lines have been identified. Six LOX-1 inactive barley landrace lines which shared the unique polymorphism in the *HvLox*-*1* structural gene region were screened from 1,153 lines (Hirota et al. 2005). A LOX-deficient mutant line was identified from a sodium azide (NaN_3_)-treated malting cultivar. Sequence analysis revealed that a C/T substitution introduced an internal stop codon into the third exon of *HvLox*-*1* (Oozeki et al. 2007). In addition, two null LOX-1 (A618 and D112) and one low LOX-1 (line G) mutant lines were also screened from generation M_3_ of a malting barley cultivar. It was believed that a splicing error and/or translational stop caused by nucleotide substitution resulted in a null LOX-1 phenotype (Patent US7420105 B2). Both artificial and natural null LOX-1 mutants are used for breeding flavor-stable malting barley for the brewing industries. Therefore, the extensive screening of natural null LOX-1 barley mutants is indispensable to high-quality barley breeding and to understanding the genetic variation of *HvLox*-*1*. In the present study, four null LOX-1 landrace lines were screened from 1,083 Chinese barley germplasm accessions. Interestingly, these four lines originated from Shan-Dong and He-Nan Provinces in the lower reaches of the Yellow River.

Sequence diversity analysis showed that there were more nucleotide polymorphisms in the intron regions (43) than in the promoter (34) and exons (23) ones of *HvLox*-*1*. Based on the 34 nucleotide polymorphisms discovered in the promoter region of *HvLox*-*1*, Chinese barley landraces and American and European barley cultivars are clearly divided into two types. SNP changes between promoters indicated that *HvLox*-*1* evolved from two distinct ancestral gene pools and independent domestication processes as previously reported (Dai et al. 2012). Notably, the second intron contained the most abundant nucleotide variations, and SNP-61 was located in this region (Table 2). A non-synonymous amino acid substitution in the third exon of the *HvLox*-*1* gene in Line G has contributed to its low LOX-1 enzyme activity phenotype (Patent US7420105 B2). Thus, the unconventional amino acid changes we discovered are likely to be functional properties of the enzyme. Since the modified rapid lipoxygenase assay is quite suitable for the qualitative detection of LOX-1 activity, the potential impact of amino acid substitutions of different haplotypes and LOX-1 activity remains unclear. A further study of a quantitative lipoxygenase assay is required to understand the relationship between non-synonymous mutation and the LOX activity.

Simple, highly reproducible, and co-dominant molecular markers derived from polymorphic sites within functional genes are essential for marker-assisted selection (Geng et al. 2012; Hirota et al. 2005). Cleaved amplified polymorphic sequence markers and allele-specific PCR fragment length polymorphism analysis (AS-PCR) are commonly used for locus-specific SNP identification (Su et al. 2011; Yang et al. 2012). In the present study, AS-PCR primers were developed for the identification of null LOX-1 barley lines. This simple method would be effective in direct marker-based selection of the null LOX-1 gene in breeding programs to create advanced null LOX-1 malting barley varieties. Finally, this work demonstrates that quality-related mutations and other valuable rare allele identifications in beer barley germplasm from different sources have considerable potential.

As a common phenomenon and an important post-transcriptional regulatory mechanism in plants, alternative splicing results in a gain or loss of protein function. The different forms of translated protein are often manifested as changes in cellular localization, stability, and activity (Barbazuk et al. 2008; Ner-Gaon et al. 2004; Wang and Brendel 2006). One of the earliest examples of alternative splicing demonstrated in plants was for Rubisco (Werneke et al. 1989). Genome-wide analyses of alternative splicing in plants were performed and reviewed in subsequent studies (Barbazuk et al. 2008; International Barley Genome Sequencing Consortium 2012; Ner-Gaon et al. 2004). Extensive alternative splicing is one notable feature of post-transcriptional processing in the barley genome. About 73 % of the intron-containing high-confidence barley genes show evidence of alternative splicing (International Barley Genome Sequencing Consortium 2012). In addition, it was reported that strict 5′- and 3′-splice site sequences of plant introns were essential to splicing factors (Reddy 2007). Thus, further investigation of the mechanism of SNP-61 inducing re-organization changes in the second intron splice site is required. It was reported that a single-nucleotide mutation altered the splicing donor site (AltD) of the fifth intron, which caused LOX deficiency in six barley lines with no significant LOX activity (Hirota et al. 2005). In the present study, a base substitution (SNP-61, C/G) in the second intron of *HvLox*-*1* was found to be associated with the null LOX-1 phenotype of the four barley landrace lines. However, further analysis of cDNA sequences identified that SNP-61 (C/G) resulted in an alternative splicing acceptor site (AltA) in the second intron of *HvLox*-*1*, which caused the LOX-1 deficiency by frame-shift and premature termination in translation (Fig. 2b, c). Since null LOX-1 haplotypes H6 and H7 were not grouped into one phylogenetic sub-tree (Fig. 3b), it was probable that a recombination event happened before and split a single mutation origin into two haplotypes. Although this finding differs from those of previous reports, given the materials used in the studies, the events of post-transcriptional processing observed in the different studies suggest that alternative splicing is an important and extensive phenomenon in barley.

## Electronic supplementary material

Below is the link to the electronic supplementary material.

Supplementary material 1 (RTF 3066 kb)

Supplementary material 2 (DOC 36 kb)

Supplementary material 3 (XLSX 13 kb)

Supplementary material 4 (XLSX 20 kb)
