# Supplementary material for: Rare allele of HvLox-1 associated with lipoxygenase activity in barley (Hordeum vulgare L.)
Source: Theor Appl Genet. 2014 Sep 12;127(10):2095–103. doi: 10.1007/s00122-014-2362-3 (PMC4180031; doi:10.1007/s00122-014-2362-3)
Supplement: Supplementary file 1 — Supplementary material 1 (RTF 3066 kb) [file 122_2014_2362_MOESM1_ESM.rtf]

             10        20        30        40         50        60        70        80         90       100
    ....|....|....|....|....|....|....|....|....|....|....|....|....|....|....|....|....|....|....|....|
H1  GCTGGTTCGTTTGCTTGTTTAACTCACATGCATCCACATATTCACATTTTCCCTAGAAAAACACAGAAAGTTCTCGTGACTTTCCTCCGTCGTCTGCAAG 
H2  ............................................................C........A..A..........T................ 
H3  ............................................................C........A..A..........T................ 
H4  ............................................................C........A..A..........T................ 
H5  ............................................................C........A..A..........T................ 
H6  ............................................................C........A..A..........T................ 
H7  ............................................................C........A..A..........T................ 

            110       120       130       140       150       160       170       180       190       200 
    ....|....|....|....|....|....|....|....|....|....|....|....|....|....|....|....|....|....|....|....|
H1  GCGCAAGTCCTGCAATATCTGATTACGCGCACACCTTGCTGCTTAGTTGGAAGATTCAAGGTGGACTGGATGAGAATGTTCATTACATGAGATCAATTTG 
H2  ..........................A................C.CA...................A.............................A... 
H3  ..........................A................C.CA...................A.............................A... 
H4  ..........................A................C.CA...................A.............................A... 
H5  ..........................A................C.CA...................A.............................A... 
H6  ..........................A................C.CA...................A.............................A... 
H7  ..........................A................C.CA...................A.............................A... 

            210       220       230       240       250       260       270       280       290       300
    ....|....|....|....|....|....|....|....|....|....|....|....|....|....|....|....|....|....|....|....|
H1  AGGCTGGGGGTCCTACCCCAACGCTCCGTTTGGATGTTTGCATTGAGAGTCTTGGCATTGCATTGGATTGAATACAAATATCACCTTGTATTGGTATTGA 
H2  ......................................................A.............................AG.A............ 
H3  ......................................................A.............................AG.A............ 
H4  ......................................................A.............................AG.A............ 
H5  ......................................................A.............................AG.A............ 
H6  ......................................................A.............................AG.A............ 
H7  ......................................................A.............................AG.A............ 

            310       320       330       340       350       360       370       380       390       400
    ....|....|....|....|....|....|....|....|....|....|....|....|....|....|....|....|....|....|....|....|
H1  GATTGAATGCCAATATTCCTGTTTGGATGTTTTGGTATCCGGAAGACGATTTCATATTGAGGTTGAATGCTAAATTCTATTTGGATGGTCAAAGTGAGGA 
H2  .........T.......T..............................G................................................... 
H3  .........T.......T..............................G................................................... 
H4  .........T.......T..............................G................................................... 
H5  .........T.......T..............................G................................................... 
H6  .........T.......T..............................G................................................... 
H7  .........T.......T..............................G................................................... 

            410       420       430       440       450       460       470       480       490       500
    ....|....|....|....|....|....|....|....|....|....|....|....|....|....|....|....|....|....|....|....|
H1  ATTAGGTTGAATGAGACTTTTCCAAGACATCAAGCAAAGAGGAGACCGAACAGGGAAGAGGGGAGCAGGCCGGTCGCTGGATCTCGCGGTGGAGGCTGGG 
H2  ....................G............A..........G.T.G................................................... 
H3  ....................G............A..........G.T.G................................................... 
H4  ....................G............A..........G.T.G................................................... 
H5  ....................G............A..........G.T.G................................................... 
H6  ....................G............A..........G.T.G................................................... 
H7  ....................G............A..........G.T.G................................................... 

            510       520       530       540       550       560       570       580       590       600
    ....|....|....|....|....|....|....|....|....|....|....|....|....|....|....|....|....|....|....|....|
H1  CGAAAGGGTGCCGCCGACCGCTGGATCTCAGGAGGGAGACGGTGTGGGAGGGTGGTGCGGGCCGTCGAAGCTTGGGGTGGTGGGTCGTCGGCCGCCGGAT 
H2  .............................G..............C..............................................T........ 
H3  .............................G..............C..............................................T........ 
H4  .............................G..............C..............................................T........ 
H5  .............................G..............C..............................................T........ 
H6  .............................G..............C..............................................T........ 
H7  .............................G..............C..............................................T........ 

            610       620       630       640       650       660       670       680       690       700
    ....|....|....|....|....|....|....|....|....|....|....|....|....|....|....|....|....|....|....|....|
H1  CTCGGGTTGGAGGCAGAGCGGGAGGAAGGGCCATCGACCACCGGATCTCGAGCTGGATGCTGGGCAGGAGGGAGTTCCTTCGACCGTCGGATCTCGCGCC 
H2  ..................................................G..............G.............C......C............. 
H3  ..................................................G..............G.............C......C............. 
H4  ..................................................G..............G.............C......C............. 
H5  ..................................................G..............G.............C......C............. 
H6  ..................................................G..............G.............C......C............. 
H7  ..................................................G..............G.............C......C............. 

            710       720       730       740       750       760       770       780       790       800
    ....|....|....|....|....|....|....|....|....|....|....|....|....|....|....|....|....|....|....|....|
H1  GAAGCTCGGGGTTGAGGGACGCCGGCCACCGGAGCCTGGGTGGAGGTGGGGGGATGGGCTACCTATGCCGCTGCCGTCGTTCCTTAGCCACACGCAGGTG 
H2  .................................................................................................... 
H3  .................................................................................................... 
H4  .................................................................................................... 
H5  .................................................................................................... 
H6  .................................................................................................... 
H7  .................................................................................................... 

            810       820       830       840       850       860       870       880       890       900
    ....|....|....|....|....|....|....|....|....|....|....|....|....|....|....|....|....|....|....|....|
H1  AGAGATCTTTTGTCTTGAGCGGGGATGAAAGGCAGGACGCTGATTTTCAATACCTAACGATGTCGAGCGCACGCCCTTCGAATTGCGGGTCGCTCGATAT 
H2  .......................T..........C.......................................................T......... 
H3  .......................T..........C.......................................................T......... 
H4  .......................T..........C.......................................................T......... 
H5  .......................T..........C.......................................................T......... 
H6  .......................T..........C.......................................................T......... 
H7  .......................T..........C.......................................................T......... 

            910       920       930       940       950       960       970       980       990       1000
    ....|....|....|....|....|....|....|....|....|....|....|....|....|....|....|....|....|....|....|....|
H1  CGAGCCTGAATGCCACGGTTGAATTTTAAAGTCATCCAAACGGTGGTAGTCAAGAAGATGAATGCCAATACTCAATATCAGGCGTGAATGTAAACATCTA 
H2  ..................................C................................................C................ 
H3  ..................................C................................................C................ 
H4  ..................................C................................................C................ 
H5  ..................................C................................................C................ 
H6  ..................................C................................................C................ 
H7  ..................................C................................................C................ 

            1010      1020      1030      1040      1050      1060      1070      1080      1090      1100
    ....|....|....|....|....|....|....|....|....|....|....|....|....|....|....|....|....|....|....|....|
H1  ATTAAACGGAGCGCAAGTGTTCTTAAAAGAAAAGAAGAAGAAAAGATTGGAGGTAGACGCTGCCTGCCATGCATGGAGTAGCAATCCTCATGAATGAAGT 
H2  ......................................................................................G............. 
H3  ......................................................................................G............. 
H4  ....................................................C............................................... 
H5  ....................................................C............................................... 
H6  ......................................................................................G............. 
H7  ....................................................C............................................... 

            1110      1120      1130      1140      1150      1160      1170      1180      1190      1200
    ....|....|....|....|....|....|....|....|....|....|....|....|....|....|....|....|....|....|....|....|
H1  CATGTGACGGCTACACACTACAAAACGCTCATTCAGCCCCATGCATGCACATGCACATGCACATGCACATGCAGTGCAGCCAAGCACCGCTCGATGGGCG 
H2  ..............................G....................................------........................... 
H3  ..............................G....................................------........................... 
H4  ........A..........................................................------........................... 
H5  ........A..........................................................------........................... 
H6  ..............................G....................................------........................... 
H7  ........A..........................................................------........................... 

            1210      1220      1230      1240      1250      1260      1270      1280      1290      1300
    ....|....|....|....|....|....|....|....|....|....|....|....|....|....|....|....|....|....|....|....|
H1  ATCACCCGTCACGGGACCGGAGCGCGCCATGCGAAGCACGAGGAGGGCACGTCACCGTCCGCGCGCAGCACGTGGAGAGCACGTCGCCGTCCGATCCATC 
H2  .................................................................................................... 
H3  .................................................................................................... 
H4  .................................................................................................... 
H5  .................................................................................................... 
H6  .................................................................................................... 
H7  .................................................................................................... 

            1310      1320      1330      1340      1350      1360      1370      1380      1390      1400
    ....|....|....|....|....|....|....|....|....|....|....|....|....|....|....|....|....|....|....|....|
H1  TCTCCAAAGCCGAGCGCCACACCACCGGGACCGGACCCGGACCGGCCTATAAATTGCCCGGACCGAGCTGCAAGCAGCTCCTCACACACACTCACGCAAC 
H2  .......................------....................................................................... 
H3  .......................------....................................................................... 
H4  .................................................................................................... 
H5  .................................................................................................... 
H6  .......................------....................................................................... 
H7  .................................................................................................... 

            1410      1420      1430      1440      1450      1460      1470      1480      1490      1500
    ....|....|....|....|....|....|....|....|....|....|....|....|....|....|....|....|....|....|....|....|
H1  ACACATCCATCTTCACTGAAAAGTGAAAAACAGTGTGCTGGTGCCATTGGTTGGAGCAGTGAAAGCGAGGAGAGGAGGCCAAGAACAAGATGCTGCTGGG 
H2  .................................................................................................... 
H3  .................................................................................................... 
H4  .................................................................................................... 
H5  .................................................................................................... 
H6  .................................................................................................... 
H7  .................................................................................................... 
                                                                                                                M  L  L  G
            1510      1520      1530      1540      1550      1560      1570      1580      1590      1600
    ....|....|....|....|....|....|....|....|....|....|....|....|....|....|....|....|....|....|....|....|
H1  AGGGCTGATCGACACCCTCACGGGGGCGAACAAGAGCGCCCGGCTCAAGGGCACGGTGGTGCTCATGCGCAAGAACGTGCTGGACCTCAACGACTTCGGC 
H2  .................................................................................................... 
H3  .................................................................................................... 
H4  .................................................................................................... 
H5  .................................................................................................... 
H6  .................................................................................................... 
H7  .................................................................................................... 
G  L  I  D  T  L  T  G  A  N  K  S  A  R  L  K  G  T  V  V  L  M  R  K  N  V  L  D  L  N  D  F  G
            1610      1620      1630      1640      1650      1660      1670      1680      1690      1700
    ....|....|....|....|....|....|....|....|....|....|....|....|....|....|....|....|....|....|....|....|
H1  GCCACCATCATCGACGGCATCGGCGAGTTCCTCGGCAAGGGCGTCACCTGCCAGCTTATCAGCTCCACCGCCGTCGACCAAGGTAATCACTACCCTCCTC 
H2  ........................................................................A........................... 
H3  ...............................................A................................C................... 
H4  ...............................................A................................C................... 
H5  ...............................................A................................C................... 
H6  ...............................................A................................C................... 
H7  ...............................................A................................C................... 
A  T  I  I  D  G  I  G  E  F  L  G  K  G  V  T  C  Q  L  I  S  S  T  A  V  D  Q
            1710      1720      1730      1740      1750      1760      1770      1780      1790      1800
    ....|....|....|....|....|....|....|....|....|....|....|....|....|....|....|....|....|....|....|....|
H1  CGGCCTTCTCCTCTGTTTACAAGATATAGTATTTCTTTCGTGTGGGCCGGCGGCCATGGATGGATGGATGTGTCTGGATCGGCTAAAGAAGATAGGATAG 
H2  ........................................................................................G........... 
H3  .................................................................................................... 
H4  .................................................................................................... 
H5  .................................................................................................... 
H6  .................................................................................................... 
H7  .................................................................................................... 
            1810      1820      1830      1840      1850      1860      1870      1880      1890      1900
    ....|....|....|....|....|....|....|....|....|....|....|....|....|....|....|....|....|....|....|....|
H1  CTAGCCCTGGCCGGTCGTCTTTACCTGAGCATGGGCATATGCCATCGAAAAAAGAGACAACAGCATGCATGCATGGTGCGCGCACCAGACCACGCAGAGC 
H2  .................................................................................................... 
H3  .................................................................................................... 
H4  .................................................................................................... 
H5  .................................................................................................... 
H6  .................................................................................................... 
H7  .................................................................................................... 
            1910      1920      1930      1940      1950      1960      1970      1980      1990      2000
    ....|....|....|....|....|....|....|....|....|....|....|....|....|....|....|....|....|....|....|....|
H1  ACCGGATGCTCGAGACAAAGCAACACAACAAGCAAGGACGACACGTCAAAAGCAACACAACAAGCAAGGACGGCACGTCAAAAGCAACACAAACCTAAAC 
H2  .................................................................................................... 
H3  .................................................................................................... 
H4  .................................................................................................... 
H5  .................................................................................................... 
H6  .................................................................................................... 
H7  .................................................................................................... 
            2010      2020      2030      2040      2050      2060      2070      2080      2090      2100
    ....|....|....|....|....|....|....|....|....|....|....|....|....|....|....|....|....|....|....|....|
H1  TAAAGCACAAAGACGTAAGAGCAAGCACACAATCAGCAGGCTATAAACAGTTGTCATCAAAAACAACGCTGGAAGAGAGAGAGAAGGAAGGAAGTAGTAG 
H2  ..............................................G..................................................... 
H3  ..............................................G..................................................... 
H4  ..............................................G..................................................... 
H5  ..............................................G..................................................... 
H6  ..............................................G..................................................... 
H7  ..............................................G..................................................... 
            2110      2120      2130      2140      2150      2160      2170      2180      2190      2200
    ....|....|....|....|....|....|....|....|....|....|....|....|....|....|....|....|....|....|....|....|
H1  CCATGAAAAATTAAATCACCGGGCGTTGCTCTTTG---------CCCAACAATTAATCAAGCAGGGTACGTGGCATGTATAGTTCTTGTAAGTAAACTAA 
H2  ..................TT...............AACTCGACT............................................C........... 
H3  ...................T...............AACTCGACT............................................C........... 
H4  ..................TT...............AACTCGACT............................................C........... 
H5  ..................TT...............AACTCGACT............................................C........... 
H6  ..................TT...............AACTCGACT............................................C........... 
H7  ..................TT...............AACTCGACT............................................C........... 
            2210      2220      2230      2240      2250      2260      2270      2280      2290      2300
    ....|....|....|....|....|....|....|....|....|....|....|....|....|....|....|....|....|....|....|....|
H1  GCATGTGATATGAGAAGGTACGTGGTGGTGCAGACAACGGCGGTCGCGGGAAGGTGGGCGCGGAGGCGGAGCTGGAGCAGTGGGTGACGAGCCTGCCGTC 
H2  .................T.................................................................................. 
H3  .................T.................................................................................. 
H4  .................T.................................................................................. 
H5  .................T.................................................................................. 
H6  .................T.................................................................................. 
H7  .................T.................................................................................. 
D  N  G  G  R  G  K  V  G  A  E  A  E  L  E  Q  W  V  T  S  L  P  S
            2310      2320      2330      2340      2350      2360      2370      2380      2390      2400 
    ....|....|....|....|....|....|....|....|....|....|....|....|....|....|....|....|....|....|....|....|
H1  GCTGACGACGGGGGAGTCCAAGTTCGGCCTCACCTTCGACTGGGAGGTGGAGAAGCTCGGGGTGCCGGGCGCCATCGTCGTCAACAACTACCACAGCTCC 
H2  .................................................................................................... 
H3  ............................................................C....................................... 
H4  ............................................................C....................................... 
H5  ............................................................C....................................... 
H6  ............................................................C....................................... 
H7  ............................................................C....................................... 
L  T  T  G  E  S  K  F  G  L  T  F  D  W  E  V  E  K  L  G  V  P  G  A  I  V  V  N  N  Y  H  S  S
            2410      2420      2430      2440      2450      2460      2470      2480      2490      2500
    ....|....|....|....|....|....|....|....|....|....|....|....|....|....|....|....|....|....|....|....|
H1  GAGTTCCTGCTTAAAACCATCACCCTCCACGACGTCCCCGGCCGCAGCGGCAACCTCACCTTCGTCGCCAACTCATGGATCTACCCCGCCGCCAACTACC 
H2  .................................................................................................... 
H3  .................................................................................................... 
H4  .................................................................................................... 
H5  .................................................................................................... 
H6  .................................................................................................... 
H7  .................................................................................................... 
E  F  L  L  K  T  I  T  L  H  D  V  P  G  R  S  G  N  L  T  F  V  A  N  S  W  I  Y  P  A  A  N  Y  
            2510      2520      2530      2540      2550      2560      2570      2580      2590      2600
    ....|....|....|....|....|....|....|....|....|....|....|....|....|....|....|....|....|....|....|....|
H1  GATACAGCCGCGTCTTCTTCGCCAACGACGTGCGTGGATTTTCCTCTACTTTCCTCTCCTTTCATTTTCACCGCCTTCGTCATTCATGGTCGATCATTAA 
H2  .C...................................T.............................................................. 
H3  .C...................................T.............................................................. 
H4  .C...................................T.............................................................. 
H5  .C...................................T.............................................................. 
H6  .C...................................T.............................................................. 
H7  .C...................................T.............................................................. 
R  Y  S  R  V  F  F  A  N  D
            2610      2620      2630      2640      2650      2660      2670      2680      2690      2700
    ....|....|....|....|....|....|....|....|....|....|....|....|....|....|....|....|....|....|....|....|
H1  GTCTTGCCAGGACAATAGATGATGAGCTAGGAGTGGTTACCACTTAGCAGTACGTACATTATTTATTCCGTGTTGGTAGAAAAGGATATGGTTTGGTGCA 
H2  ..............C...............C...............A........................C............................ 
H3  ..............C...............C........................................C............................ 
H4  ..............C.......................................................................A............. 
H5  ..............C.......................................................................A............. 
H6  ..............C.......................................................................A............. 
H7  ..............C.......................................................................A............. 
            2710      2720      2730      2740      2750      2760      2770      2780      2790      2800
    ....|....|....|....|....|....|....|....|....|....|....|....|....|....|....|....|....|....|....|....|
H1  GATCGACACAAGATTGAATGAAAGTTGCACCGTGGCACCGTGGCAGCGTGGTAGGTGAAAATAACTGTTGCACGGATCCACCCACATGATTGTTTTCATG 
H2  ......................................--------....................................................A. 
H3  .................................................................................................... 
H4  ......................................--------...................................................... 
H5  ......................................--------...................................................... 
H6  ......................................--------...................................................... 
H7  ......................................--------...................................................... 

            2810      2820      2830      2840      2850      2860      2870      2880      2890      2900
    ....|....|....|....|....|....|....|....|....|....|....|....|....|....|....|....|....|....|....|....|
H1  AATAAACTTTTTAAGGATGTGTCTAGCCACATCTAGATGCATGTCACATAATTATTGCATACCAAAACGATTAAATTAAGCATAAAAAGAAAAGG-AAAA 
H2  ...........C...................................................................................-.... 
H3  ...............................................................................................-.... 
H4  ...............................................................................................A.... 
H5  ...............................................................................................A.... 
H6  ...............................................................................................A.... 
H7  ...............................................................................................A.... 

            2910      2920      2930      2940      2950      2960      2970      2980      2990      3000
    ....|....|....|....|....|....|....|....|....|....|....|....|....|....|....|....|....|....|....|....|
H1  AAATACTCACATATCTCGACGTAAGATCAATGATATAGTATTTA--------------GATATGCAATATTTATCTTACATCTAAACCTTTCTTCATTCC 
H2  ..................................G.........--------------..........................G............... 
H3  ............................................--------------..........................G............... 
H4  .................C.G........................TGATATAGTATTTA..........................G............... 
H5  .................C.G........................TGATATAGTATTTA..........................G............... 
H6  .................C.G........................TGATATAGTATTTA..........................G............... 
H7  .................C.G........................TGATATAGTATTTA..........................G............... 

            3010      3020      3030      3040      3050      3060      3070      3080      3090      3100
    ....|....|....|....|....|....|....|....|....|....|....|....|....|....|....|....|....|....|....|....|
H1  TAAATATAAGACATTTGTAAGATTTCACTATGGACAACATACGAAACAAAATCAGTGGATC-------------------TCTCTATGCATTCATTATGT 
H2  ...........................................G.................-------------------.................... 
H3  .........................T.................G........G........TACATCCTAAAA---TATG...A..........C..... 
H4  ...T............TA.......T......A..........G...-....G........TACATTTTAAAATACTATG...A...C......C..... 
H5  ...T............TA.......T......A..........G...-....G........TACATTTTAAAATACTATG...A...C......C..... 
H6  ...T............TA.......T......A..........G...-....G........TACATTTTAAAATACTATG...A...C......C..... 
H7  ...T............TA.......T......A..........G...-....G........TACATTTTAAAATACTATG...A...C......C..... 

            3110      3120      3130      3140      3150      3160      3170      3180      3190      3200
    ....|....|....|....|....|....|....|....|....|....|....|....|....|....|....|....|....|....|....|....|
H1  AGTCTATAATAAAATCTTTAAAAGATCGTATATTTTGCAACGGAGGGAGTAAAACATAACTTTTTAATAGTAATGTTGCACGGCTCCACACTCGCAGACG 
H2  ..............AT...........A........................................................................ 
H3  ...........................A........................................................................ 
H4  ................A..-.......A........................................................................ 
H5  ................A..-.......A........................................................................ 
H6  ................A..-.......A....................................................G................... 
H7  ................A..-.......A....................................................G................... 
   T 


            3210      3220      3230      3240      3250      3260      3270      3280      3290      3300
    ....|....|....|....|....|....|....|....|....|....|....|....|....|....|....|....|....|....|....|....|
H1  TACCTGCCGAGCCAGATGCCGGCGGCGCTGAAGCCGTACCGCGACGACGAGCTCCGGAACCTGCGTGGCGACGACCAGCAGGGCCCGTACCAGGAGCACG 
H2  ...................................A................................................................ 
H3  ...................................A................................................................ 
H4  .........................................G.......................................................... 
H5  .........................................G.......................................................... 
H6  .........................................G.......................................................... 
H7  .........................................G.......................................................... 
Y  L  P  S  Q  M  P  A  A  L  K  P  Y  R  D  D  E  L  R  N  L  R  G  D  D  Q  Q  G  P  Y  Q  E  H  

            3310      3320      3330      3340      3350      3360      3370      3380      3390      3400
    ....|....|....|....|....|....|....|....|....|....|....|....|....|....|....|....|....|....|....|....|
H1  ACCGCATCTACCGCTACGACGTCTACAACGACCTCGGCGAGGGCCGCCCCATCCTCGGCGGCAACTCCGACCACCCTTACCCGCGCCGCGGCCGCACGGA 
H2  .....................................................................................T.............G 
H3  .....G.............................................................................................G 
H4  .....G.............................................................................................G 
H5  .....G.............................................................................................G 
H6  .....G.............................................................................................G 
H7  .....G.............................................................................................G 
D  R  I  Y  R  Y  D  V  Y  N  D  L  G  E  G  R  P  I  L  G  G  N  S  D  H  P  Y  P  R  R  G  R  T  E

            3410      3420      3430      3440      3450      3460      3470      3480      3490      3500
    ....|....|....|....|....|....|....|....|....|....|....|....|....|....|....|....|....|....|....|....|
H1  GCGCAAGCCCAACGCCAGCGACCCGAGCCTGGAGAGCCGGCTGTCGCTGCTGGAGCAGATCTACGTGCCGCGGGACGAGAAGTTCGGCCACCTCAAGACG 
H2  .................................................................................................... 
H3  .................................................................................................... 
H4  .................................................................................................... 
H5  .................................................................................................... 
H6  .................................................................................................... 
H7  .................................................................................................... 
      R  K  P  N  A  S  D  P  S  L  E  S  R  L  S  L  L  E  Q  I  Y  V  P  R  D  E  K  F  G  H  L  K  T 

            3510      3520      3530      3540      3550      3560      3570      3580      3590      3600
    ....|....|....|....|....|....|....|....|....|....|....|....|....|....|....|....|....|....|....|....|
H1  TCCGACTTCCTGGGCTACTCCATCAAGGCCATCACGCAGGGCATCCTGCCGGCCGTGCGCACCTACGTGGACACCACCCCCGGCGAGTTCGACTCCTTCC 
H2  .................................................................................................... 
H3  .................................................................................................... 
H4  .................................................................................................... 
H5  .................................................................................................... 
H6  .................................................................................................... 
H7  .................................................................................................... 
S  D  F  L  G  Y  S  I  K  A  I  T  Q  G  I  L  P  A  V  R  T  Y  V  D  T  T  P  G  E  F  D  S  F  

            3610      3620      3630      3640      3650      3660      3670      3680      3690      3700
    ....|....|....|....|....|....|....|....|....|....|....|....|....|....|....|....|....|....|....|....|
H1  AGGACATCATCAACCTCTATGAGGGCGGCATCAAGCTGCCCAAGGTGGCCGCCCTGGAGGAGCTCCGTAAGCAGTTCCCGCTCCAGCTCATCAAGGACCT 
H2  .................................................................................................... 
H3  .................................................................................................... 
H4  .................................................................................................... 
H5  .................................................................................................... 
H6  .................................................................................................... 
H7  .................................................................................................... 
Q  D  I  I  N  L  Y  E  G  G  I  K  L  P  K  V  A  A  L  E  E  L  R  K  Q  F  P  L  Q  L  I  K  D  L

            3710      3720      3730      3740      3750      3760      3770      3780      3790      3800
    ....|....|....|....|....|....|....|....|....|....|....|....|....|....|....|....|....|....|....|....|
H1  CCTCCCCGTCGGCGGCGACTCCCTGCTTAAGCTCCCCGTGCCCCACATCATCCAGGAGAACAAGCAGGCGTGGAGGACCGACGAGGAGTTCGCACGGGAG 
H2  .................................................................................................... 
H3  .................................................................................................... 
H4  .................................................................................................... 
H5  .................................................................................................... 
H6  .................................................................................................... 
H7  .................................................................................................... 
L  P  V  G  G  D  S  L  L  K  L  P  V  P  H  I  I  Q  E  N  K  Q  A  W  R  T  D  E  E  F  A  R  E 

            3810      3820      3830      3840      3850      3860      3870      3880      3890      3900
    ....|....|....|....|....|....|....|....|....|....|....|....|....|....|....|....|....|....|....|....|
H1  GTGCTCGCCGGCGTCAACCCGGTCATGATCACGCGTCTCACGGTGAGTCAGCGATTATTTGTTCATTGTGTGTGTATGGTGTCCATGGTGAGAAAGTGCA            
H2  ..................................................A...............--..........A..................... 
H3  ..................................................A...............--..........A..................... 
H4  .................................................................................................... 
H5  .................................................................................................... 
H6  .................................................................................................... 
H7  .................................................................................................... 
V  L  A  G  V  N  P  V  M  I  T  R  L  T


            3910      3920      3930      3940      3950      3960      3970      3980      3990      4000
    ....|....|....|....|....|....|....|....|....|....|....|....|....|....|....|....|....|....|....|....|
H1  GATCTTGATTTGCGTTGGGTCGCATGCACGCATGCTGCATGCATGCAGGAGTTCCCGCCAAAAAGTAGTCTGGACCCTAGCAAGTTTGGTGACCACACCA 
H2  .................................................................................................... 
H3  .................................................................................................... 
H4  .................................................................................................... 
H5  .................................................................................................... 
H6  .................................................................................................... 
H7  .................................................................................................... 
E  F  P  P  K  S  S  L  D  P  S  K  F  G  D  H  T  

            4010      4020      4030      4040      4050      4060      4070      4080      4090      4100
    ....|....|....|....|....|....|....|....|....|....|....|....|....|....|....|....|....|....|....|....|
H1  GCACCATCACGGCGGAGCACATAGAGAAGAACCTCGAGGGCCTCACGGTGCAGCAGGTAATTGGTCCAAGCCATCGACATCAACTATGATTTACCTAGGA 
H2  .................................................................................................... 
H3  .................................................................................................... 
H4  .............................................................................------------........... 
H5  .............................................................................------------........... 
H6  .............................................................................------------........... 
H7  .............................................................................------------........... 
S  T  I  T  A  E  H  I  E  K  N  L  E  G  L  T  V  Q  Q

            4110      4120      4130      4140      4150      4160      4170      4180      4190      4200
    ....|....|....|....|....|....|....|....|....|....|....|....|....|....|....|....|....|....|....|....|
H1  GTAATTGGTAGCTGTAGATAATTTGGCTTCGTTGCAATTAATTTGATGCTGGCCGATCAAGTGATCGTATTGGGTTTGAAATTTGCAGGCGCTGGAAAGC 
H2  .................................................................................................... 
H3  .................................................................................................... 
H4  .................................................................................................... 
H5  .................................................................................................... 
H6  .................................................................................................... 
H7  .................................................................................................... 
A  L  E  S 

            4210      4220      4230      4240      4250      4260      4270      4280      4290      4300
    ....|....|....|....|....|....|....|....|....|....|....|....|....|....|....|....|....|....|....|....|
H1  AACAGGCTGTACATCCTTGATCACCATGACCGGTTCATGCCGTTCCTGATCGACGTCAACAACCTGCCCGGCAACTTCATCTACGCCACGAGGACCCTCT 
H2  .................................................................................................... 
H3  .................................................................................................... 
H4  .................................................................................................... 
H5  .................................................................................................... 
H6  .................................................................................................... 
H7  .................................................................................................... 
N  R  L  Y  I  L  D  H  H  D  R  F  M  P  F  L  I  D  V  N  N  L  P  G  N  F  I  Y  A  T  R  T  L  

            4310      4320      4330      4340      4350      4360      4370      4380      4390      4400
    ....|....|....|....|....|....|....|....|....|....|....|....|....|....|....|....|....|....|....|....|
H1  TCTTCCTGCGCGGCGACGGCAGGCTCACGCCGCTCGCCATCGAGCTGAGCGAGCCCATCATCCAGGGCGGCCTTACCACGGCCAAGAGCAAGGTTTACAC 
H2  .................................................................................................... 
H3  .................................................................................................... 
H4  .................................................................................................T.. 
H5  .................................................................................................T.. 
H6  .................................................................................................T.. 
H7  .................................................................................................T.. 
F  F  L  R  G  D  G  R  L  T  P  L  A  I  E  L  S  E  P  I  I  Q  G  G  L  T  T  A  K  S  K  V  Y  T

            4410      4420      4430      4440      4450      4460      4470      4480      4490      4500
    ....|....|....|....|....|....|....|....|....|....|....|....|....|....|....|....|....|....|....|....|
H1  GCCGGTGCCCAGCGGCTCCGTCGAAGGCTGGGTGTGGGAGCTCGCCAAGGCCTACGTCGCCGTCAATGACTCCGGGTGGCACCAGCTCGTCAGCCACTGG 
H2  .................................................................................................... 
H3  .................................................................................................... 
H4  .................................................................................................... 
H5  .................................................................................................... 
H6  .................................................................................................... 
H7  .................................................................................................... 
P  V  P  S  G  S  V  E  G  W  V  W  E  L  A  K  A  Y  V  A  V  N  D  S  G  W  H  Q  L  V  S  H  W

            4510      4520      4530      4540      4550      4560      4570      4580      4590      4600
    ....|....|....|....|....|....|....|....|....|....|....|....|....|....|....|....|....|....|....|....|
H1  TACGTTCTCCACGGTCGATGTGATTCAGTCAGTCGATGCACAACAACTGATCGAAATATGATTGATTGAAACGCGCAGGCTGAACACTCACGCGGTGATG 
H2  .................................................................................................... 
H3  .................................................................................................... 
H4  ....................C............................................................................... 
H5  ....................C............................................................................... 
H6  ....................C............................................................................... 
H7  ....................C............................................................................... 
L  N  T  H  A  V  M  


            4610      4620      4630      4640      4650      4660      4670      4680      4690      4700
    ....|....|....|....|....|....|....|....|....|....|....|....|....|....|....|....|....|....|....|....|
H1  GAGCCGTTCGTGATCTCGACGAACCGGCACCTTAGCGTGACGCACCCGGTGCACAAGCTGCTGAGCCCGCACTACCGCGACACCATGACCATCAACGCGC 
H2  .................................................................................................... 
H3  .................................................................................................... 
H4  .................................................................................................... 
H5  .................................................................................................... 
H6  .................................................................................................... 
H7  .................................................................................................... 
E  P  F  V  I  S  T  N  R  H  L  S  V  T  H  P  V  H  K  L  L  S  P  H  Y  R  D  T  M  T  I  N  A  

            4710      4720      4730      4740      4750      4760      4770      4780      4790      4800
    ....|....|....|....|....|....|....|....|....|....|....|....|....|....|....|....|....|....|....|....|
H1  TGGCGCGGCAGACGCTCATCAACGCCGGCGGCATCTTCGAGATGACGGTGTTCCCGGGCAAGTTCGCGTTGGGGATGTCGGCCGTGGTGTACAAGGACTG 
H2  ........................................A........................................................... 
H3  ................................................................T...C......................T........ 
H4  ................................................................T...C......................T........ 
H5  ................................................................T...C......................T........ 
H6  ................................................................T...C......................T........ 
H7  ................................................................T...C......................T........ 
L  A  R  Q  T  L  I  N  A  G  G  I  F  E  M  T  V  F  P  G  K  F  A  L  G  M  S  A  V  V  Y  K  D  W  

            4810      4820      4830      4840      4850      4860      4870      4880      4890      4900
    ....|....|....|....|....|....|....|....|....|....|....|....|....|....|....|....|....|....|....|....|
H1  GAAGTTCACCGAGCAGGGACTGCCGGACGATCTCATCAAGAGGTAC---GTACCTGGTAAATGTTATGAATGTGTAAAACAAATTGGGCGTCTCGCTCAC 
H2  ..............................................---................................................... 
H3  ........................C.....................---T..A.AT.C.TGCAG.GA......AA...CA....GA.AATGTGGTGA.C. 
H4  ........................C.....................---T..A.AT.C.TGCAG.GA......AA...CA....GA.AATGTGGTGA.C. 
H5  ........................C.....................TACT.GA.AT.C.TGCAG.GA......AA...CA....GA.AATGTGGTGA.C. 
H6  ..............................................---................................................... 
H7  ........................C.....................---T..A.AT.C.TGCAG.GA......AA...CA....GA.AATGTGGTGA.C. 
K  F  T  E  Q  G  L  P  D  D  L  I  K  R

            4910      4920      4930      4940      4950      4960      4970      4980      4990      5000
    ....|....|....|....|....|....|....|....|....|....|....|....|....|....|....|....|....|....|....|....|
H1  TGACAGGAACGTGGTAAAAAAAA--------------TGCAGGGGCATGGCGGTGGAGGACCCGTCGAGCCCGTACAAGGTGCGGTTGCTGGTGTCGGAC 
H2  .......................--------------............................................................... 
H3  GA.ACA...ACAAAA.CC...TGCTTGCGTGCGTGCG................................................C.............. 
H4  GA.ACA...ACAAAA.CC...TGCTTGCGTGCGTGCG................................................C.............. 
H5  GA.ACA...ACAAAA.TC...TGCTTGCGTGCGTGCG................................................C.............. 
H6  .......................--------------............................................................... 
H7  GA.ACA...ACAAAA.CC...TGCTTGCGTGCGTGCG................................................C.............. 
G  M  A  V  E  D  P  S  S  P  Y  K  V  R  L  L  V  S  D  

            5010      5020      5030      5040      5050      5060      5070      5080      5090      5100
    ....|....|....|....|....|....|....|....|....|....|....|....|....|....|....|....|....|....|....|....|
H1  TACCCGTACGCGGCGGACGGGCTGGCGATCTGGCACGCCATTGAGCAGTACGTGAGCGAGTACCTGGCCATCTACTACCCGAACGACGGCGTGCTGCAGG 
H2  .................................................................................................... 
H3  .................................................................................................... 
H4  .................................................................................................... 
H5  ..............A..................................................................................... 
H6  .................................................................................................... 
H7  .................................................................................................... 
Y  P  Y  A  A  D  G  L  A  I  W  H  A  I  E  Q  Y  V  S  E  Y  L  A  I  Y  Y  P  N  D  G  V  L  Q  

            5110      5120      5130      5140      5150      5160      5170      5180      5190      5200
    ....|....|....|....|....|....|....|....|....|....|....|....|....|....|....|....|....|....|....|....|
H1  GCGATACGGAGGTGCAGGCGTGGTGGAAGGAGACGCGCGAGGTCGGGCACGGCGACCTCAAGGACGCCCCATGGTGGCCCAAGATGCAAAGTGTGCCGGA 
H2  .................................................................................................... 
H3  ..........................................................................................C......... 
H4  ..........................................................................................C......... 
H5  .................................................................................................... 
H6  .................................................................................................... 
H7  ..........................................................................................C......... 
G  D  T  E  V  Q  A  W  W  K  E  T  R  E  V  G  H  G  D  L  K  D  A  P  W  W  P  K  M  Q  S  V  P  E  

            5210      5220      5230      5240      5250      5260      5270      5280      5290      5300
    ....|....|....|....|....|....|....|....|....|....|....|....|....|....|....|....|....|....|....|....|
H1  GCTGGCCAAGGCGTGCACCACCATCATCTGGATCGGGTCGGCGCTGCATGCGGCAGTCAACTTCGGGCAGTACCCCTACGCGGGGTTCCTCCCGAACCGG 
H2  .................................................................................................... 
H3  .................................................................................................... 
H4  .................................................................................................... 
H5  .................................................................................................... 
H6  .................................................................................................... 
H7  .................................................................................................... 
L  A  K  A  C  T  T  I  I  W  I  G  S  A  L  H  A  A  V  N  F  G  Q  Y  P  Y  A  G  F  L  P  N  R  


            5310      5320      5330      5340      5350      5360      5370      5380      5390      5400 
    ....|....|....|....|....|....|....|....|....|....|....|....|....|....|....|....|....|....|....|....|
H1  CCGACGGTGAGCCGGCGCCGCATGCCGGAGCCCGGCACGGAGGAGTACGCGGAGCTGGAGCGCGACCCGGAGCGGGCCTTCATCCACACCATCACGAGCC 
H2  .......................................C.........T.................................................. 
H3  .................................................................................................... 
H4  .................................................................................................... 
H5  .................................................................................................... 
H6  .......................................C.........T.................................................. 
H7  .................................................................................................... 
P  T  V  S  R  R  R  M  P  E  P  G  T  E  E  Y  A  E  L  E  R  D  P  E  R  A  F  I  H  T  I  T  S  
            5410      5420      5430      5440      5450      5460      5470      5480      5490      5500
    ....|....|....|....|....|....|....|....|....|....|....|....|....|....|....|....|....|....|....|....|
H1  AGATCCAGACCATCATCGGCGTGTCGCTGCTGGAGGTGCTGTCGAAGCACTCCTCCGACGAGCTGTACCTCGGGCAGCGGGACACGCCGGAGTGGACCTC 
H2  .................................................................................................... 
H3  .................................................................................................... 
H4  .................................................................................................... 
H5  .................................................................................................... 
H6  .................................................................................................... 
H7  .................................................................................................... 
Q  I  Q  T  I  I  G  V  S  L  L  E  V  L  S  K  H  S  S  D  E  L  Y  L  G  Q  R  D  T  P  E  W  T  S  
            5510      5520      5530      5540      5550      5560      5570      5580      5590      5600
    ....|....|....|....|....|....|....|....|....|....|....|....|....|....|....|....|....|....|....|....|
H1  GGACCCAAAGGCCCTGGAGGTGTTCAAGCGGTTCAGCGACCGGCTGGTGGAGATCGAGAGCAAGGTGGTGGGCATGAACCATGACCCGGAGCTCAAGAAC 
H2  .................................................................................................... 
H3  .................................................................................................... 
H4  .................................................................................................... 
H5  .....................................................C.............................................. 
H6  .................................................................................................... 
H7  .................................................................................................... 
D  P  K  A  L  E  V  F  K  R  F  S  D  R  L  V  E  I  E  S  K  V  V  G  M  N  H  D  P  E  L  K  N  
            5610      5620      5630      5640      5650      5660      5670      5680      5690      5700
    ....|....|....|....|....|....|....|....|....|....|....|....|....|....|....|....|....|....|....|....|
H1  CGCAACGGCCCGGCTAAGTTTCCCTACATGCTGCTCTACCCCAACACCTCCGACCACAAGGGCGCCGCTGCCGGGCTTACCGCCAAGGGCATCCCCAACA 
H2  ............C.C.....C............................................................................... 
H3  .................................................................................................... 
H4  .................................................................................................... 
H5  ............C.C.....C............................................................................... 
H6  ............C.C.....C............................................................................... 
H7  .................................................................................................... 
R  N  G  P  A  K  F  P  Y  M  L  L  Y  P  N  T  S  D  H  K  G  A  A  A  G  L  T  A  K  G  I  P  N  
            5710      5720      5730      5740      5750      5760      5770      5780      5790      5800
    ....|....|....|....|....|....|....|....|....|....|....|....|....|....|....|....|....|....|....|....|
H1  GCATCTCCATCTAATCTAAGCCATCGGCAACCATGGATGAATAAAGGGCGTTCGCCACGTACGAAACTTGTCGAGAGATTGGTGTA--GTGTGTGTCTGT 
H2  ......................................................................................GT............ 
H3  ................................................T.....................................GT............ 
H4  ................................................T.....................................GT............ 
H5  ......................................................................................GT............ 
H6  ......................................................................................GT............ 
H7  ................................................T.....................................GT............ 
S  I  S  I  *
            5810      5820      5830      5840      5850      5860      5870      5880      5890      5900
    ....|....|....|....|....|....|....|....|....|....|....|....|....|....|....|....|....|....|....|....|
H1  GACAGTACTATGTCAGCAGTTGCTCTTTAAGCCGAATAAATAAAGCAGATTTGCTTCCACTGGAATTGTGTCAGTCATGGCATTTACTATTGTTTGAAAT 
H2  .................................................................................................... 
H3  .................................................................................................... 
H4  .................................................................................................... 
H5  ........................-........................................................................... 
H6  .................................................................................................... 
H7  .................................................................................................... 

            5910      5920      5930      5940
    ....|....|....|....|....|....|....|....|....|.
H1  GGATGAAAAGAAATGCCCCATCCGTTTTTAAGCGGAACGAATTGCT 
H2  .............................................. 
H3  .............................................. 
H4  .............................................. 
H5  .............................................. 
H6  .............................................. 
H7  .............................................. 

Supplementary Figure 1. Alignment of the seven haplotype sequences of HvLox-1. 
Cyan indicates the exons of HvLox-1. The corresponding amino acid sequence encoded by H1 were shown below. Amino acid changes which caused by SNPs were indicated with underline and blue (synonymous) and red (non-synonymous) characters.
