# Supplementary material for: Rare allele of HvLox-1 associated with lipoxygenase activity in barley (Hordeum vulgare L.)
Source: Theor Appl Genet. 2014 Sep 12;127(10):2095–103. doi: 10.1007/s00122-014-2362-3 (PMC4180031; doi:10.1007/s00122-014-2362-3)
Supplement: Supplementary file 2 — Supplementary material 2 (DOC 36 kb) [file 122_2014_2362_MOESM2_ESM.doc]

**Table S1. PCR primers used in this experiment.**

| Name | Forward (5’-3’) | Purpose |
| --- | --- | --- |
| Lox1.1F | GCTGGTTCGTTTGCTTGTTT | Genomic DNA sequencing |
| Lox1.1R | CTCTCCTCGCTTTCACTGCT |
| Lox1.2F | GTCCGATCCATCTCTCCAAA |
| Lox1.2R | TCGATCTGCACCAAACCATA |
| Lox1.3F | CTTTCATTTTCACCGCCTTC |
| Lox1.3R | GGCACGTAGATCTGCTCCA |
| Lox1.4F | CGCTACGACGTCTACAACGA |
| Lox1.4R | GCGCGTTTCAATCAATCATA |
| Lox1.5F | GTACGTTCTCCACGGTCGAT |
| Lox1.5R | AGCAATTCGTTCCGCTTAAA |
| Lox1.FL.1F | AGCAGTGAAAGCGAGGAGAG | cDNA sequencing |
| Lox1.FL.1R | TGATGGAGTAGCCCAGGAAG |
| Lox1.FL.2F | CGCCAACTCATGGATCTACC |
| Lox1.FL.2R | GAACGCCCTTTATTCATCCA |
| RNF | AAAACATAACTTTTTAATAGTAATGTTGCA**C** | SNP-61 (C) identification |
| RNR | CACTAGTGAT TCTGCGAGTGTGGAGCC**C** | SNP-61 (G) identification |
